# Supplementary material for: Pyruvate Dehydrogenase Kinase 1 inhibition mediated oxidative phosphorylation enhancement in cartilage promotes osteoarthritis progression
Source: BMC Musculoskelet Disord. 2023 Jul 20;24:597. doi: 10.1186/s12891-023-06585-6 (PMC10357736; doi:10.1186/s12891-023-06585-6)
Supplement: Supplementary file 1 — Additional file 1. [file 12891_2023_6585_MOESM1_ESM.zip › BP.Gsea.1653623667859/GOBP_GLOMERULAR_MESANGIUM_DEVELOPMENT.html]

Details for gene set GOBP\_GLOMERULAR\_MESANGIUM\_DEVELOPMENT[GSEA]

|  || Dataset | 55457\_collapsed\_to\_symbols.cls.cls #OA\_versus\_normal.cls.cls #OA\_versus\_normal\_repos |
| Phenotype | cls.cls#OA\_versus\_normal\_repos |
| Upregulated in class | normal |
| GeneSet | GOBP\_GLOMERULAR\_MESANGIUM\_DEVELOPMENT |
| Enrichment Score (ES) | -0.73173964 |
| Normalized Enrichment Score (NES) | -2.1446674 |
| Nominal p-value | 0.0 |
| FDR q-value | 0.0055129444 |
| FWER p-Value | 0.032 |
Table: GSEA Results Summary

  

Fig 1: Enrichment plot: GOBP\_GLOMERULAR\_MESANGIUM\_DEVELOPMENT      
 Profile of the Running ES Score & Positions of GeneSet Members on the Rank Ordered List

  

| SYMBOL | TITLE | RANK IN GENE LIST | RANK METRIC SCORE | RUNNING ES | CORE ENRICHMENT || 1 | PDGFD | platelet derived growth factor D [Source:HGNC Symbol;Acc:HGNC:30620] | 3850 | 0.171 | -0.2719 | No |
| 2 | CD34 | CD34 molecule [Source:HGNC Symbol;Acc:HGNC:1662] | 4341 | 0.125 | -0.2903 | No |
| 3 | ACTA2 | "actin alpha 2, smooth muscle [Source:HGNC Symbol;Acc:HGNC:130]" | 4490 | 0.114 | -0.2838 | No |
| 4 | WT1 | WT1 transcription factor [Source:HGNC Symbol;Acc:HGNC:12796] | 5452 | 0.037 | -0.3526 | No |
| 5 | PDGFRB | platelet derived growth factor receptor beta [Source:HGNC Symbol;Acc:HGNC:8804] | 6914 | -0.073 | -0.4545 | No |
| 6 | SERPINB7 | serpin family B member 7 [Source:HGNC Symbol;Acc:HGNC:13902] | 7679 | -0.133 | -0.4928 | No |
| 7 | BMP4 | bone morphogenetic protein 4 [Source:HGNC Symbol;Acc:HGNC:1071] | 7816 | -0.142 | -0.4809 | No |
| 8 | BMP7 | bone morphogenetic protein 7 [Source:HGNC Symbol;Acc:HGNC:1074] | 9544 | -0.278 | -0.5711 | No |
| 9 | IL6R | interleukin 6 receptor [Source:HGNC Symbol;Acc:HGNC:6019] | 11615 | -0.519 | -0.6499 | Yes |
| 10 | EGR1 | early growth response 1 [Source:HGNC Symbol;Acc:HGNC:3238] | 12052 | -0.607 | -0.5880 | Yes |
| 11 | CFLAR | CASP8 and FADD like apoptosis regulator [Source:HGNC Symbol;Acc:HGNC:1876] | 12079 | -0.614 | -0.4932 | Yes |
| 12 | PDGFA | platelet derived growth factor subunit A [Source:HGNC Symbol;Acc:HGNC:8799] | 12104 | -0.621 | -0.3971 | Yes |
| 13 | FOXC2 | forkhead box C2 [Source:HGNC Symbol;Acc:HGNC:3801] | 12218 | -0.650 | -0.3034 | Yes |
| 14 | PDGFB | platelet derived growth factor subunit B [Source:HGNC Symbol;Acc:HGNC:8800] | 12389 | -0.704 | -0.2055 | Yes |
| 15 | NOTCH1 | notch receptor 1 [Source:HGNC Symbol;Acc:HGNC:7881] | 12555 | -0.776 | -0.0960 | Yes |
| 16 | GPR4 | G protein-coupled receptor 4 [Source:HGNC Symbol;Acc:HGNC:4497] | 12561 | -0.778 | 0.0263 | Yes |
Table: GSEA details [plain text format]

  

Fig 2: GOBP\_GLOMERULAR\_MESANGIUM\_DEVELOPMENT      
 Blue-Pink O' Gram in the Space of the Analyzed GeneSet

  

Fig 3: GOBP\_GLOMERULAR\_MESANGIUM\_DEVELOPMENT: Random ES distribution      
 Gene set null distribution of ES for **GOBP\_GLOMERULAR\_MESANGIUM\_DEVELOPMENT**
